# Complementary Feeding in Preterm Infants: Where Do We Stand?

**DOI:** 10.3390/nu12051259

**Published:** 2020-04-29

**Authors:** Maria Elisabetta Baldassarre, Maria Lorella Giannì, Antonio Di Mauro, Fabio Mosca, Nicola Laforgia

**Affiliations:** 1 Department of Biomedical Science and Human Oncology, Section of Neonatology and NICU, University “Aldo Moro”, 70100 Bari, Italy; 2 Fondazione IRCCS Ca’ Granda Ospedale Maggiore Policlinico, NICU, Via Commenda 12, 20122 Milan, Italy; 3 Department of Clinical Sciences and Community Health, University of Milan, Via Commenda 19, 20122 Milan, Italy; 4 Italian Society of Neonatology, Corso Venezia 8, 20121 Milan, Italy

Currently, about 15 million preterm births occur annually worldwide; over 500,000 in Europe and 32,000 in Italy, accounting for 7–11% of total births, with the highest incidence in low-income states. Most babies are born before 32 weeks of gestation, whereas 1.58% are born earlier [1].

Great effort has been made to decrease the burden of mortality and morbidity of prematurity [2]. Preterm infants experience the impaired development of the structure and function of key organs and systems due to the interruption of the physiological intrauterine organogenesis with consequent exposure to the extrauterine environment at a time when organ plasticity is exceptionally high. Preterm infants are at increased risk of adverse health outcomes, further exacerbated by the frequent association of different co-morbidities [2,3,4].

Early nutrition and growth are key contributors to the modulation of both short and long-term infant health outcomes [5]. Preterm infants have different nutritional needs to their term peers in terms of energy, macronutrients and micronutrients intake [6]. Besides, they frequently develop significant postnatal growth retardation [7] with altered body composition [8,9]. Preterm infants develop a relative reduction in fat free mass with increased adiposity, which, respectively, may contribute to adverse neuro and metabolic outcomes [8,9,10,11]. Accordingly, both the prevention and recovery of any nutritional deficits accounting for growth pattern and body composition alterations should be a priority for the nutritional care of infants born preterm.

The introduction of solid foods (thereafter referred to as weaning) is associated with major changes in both macronutrients and micronutrients intake, with the risk of nutritional deficits or excesses for infants undergoing a rapid growth and development during this period of life [12]. Yet, surprisingly, relatively little attention has been paid to defining both the ideal age and the detailed content of weaning and to their future possible effects on later health and development [13]. No evidence-based guidelines are available regarding the most appropriate time and method of weaning preterm infants—a much-debated issue, partly because of the postnatal cumulative nutritional deficits reported in this very vulnerable population [14].

In 1974 and 1980, the English Department of Health suggested that “few infants should require solids before 3 months and most by 6 months” [15,16]. Then, in 1994, the Committee on Medical Aspects of Food and Nutrition Policy recommended the introduction of solid foods between 5 and 8 months of age, but the individual differences among infants, in terms of either acquired development milestones or specific nutritional needs, were not evaluated [17]. The Joint Consensus Statement on weaning of preterm infants in 2008 suggested “preterm infants should be considered for weaning between 5 and 8 months of uncorrected age to ensure that sensitive periods for the acceptance of solids are not missed and to allow development of appropriate feeding skills” [18]. In 2012, Palmer and Makrides, on the basis of limited available evidence, concluded that starting weaning at three months of corrected age could be appropriate for the majority of preterm infants, including those of lowest gestational ages [19]. Moreover, weaning too early (before 16 weeks of chronological age) may cause an increased risk of allergy and anemia whereas a delayed weaning (after 7 to 10 months of chronological age) may lead to the development of avoidance feeding behavior [19]. Norris et al. evaluated current infant feeding practices among caregivers of preterm infants: two hundred fifty-three infants born preterm were recruited in southeast England over a 2-year period [20]. They reported that the introduction of solid foods varied widely, and that compliance with the few existing general recommendations on weaning was poor [20]. In accordance with these findings, an Italian study demonstrated that, in the absence of a general consensus, primary-care pediatricians have very different approaches to the weaning of healthy preterm infants [21].

Although the early introduction of weaning foods has been suggested for promoting weight gain and recovery of nutritional deficits [22], there is general concern regarding the association between weight gain and the increased risk of obesity and metabolic syndrome later in life [23]. Previous studies have reported inconsistent findings regarding the relationship between the start of weaning and growth, probably because preterm infants of different gestational ages and birth weights were included. In a blinded randomized clinical trial including preterm infants <37 weeks of gestation, Marriot et al. found, in infants weaned at 14.9 weeks postnatal age (intervention group) compared to infants weaned at 17.8 weeks postnatal age (control group), between term and 12 months of corrected age, a greater increase in length growth velocity and higher mean hemoglobin and serum iron levels at 6 months of corrected age [24]. Spiegler et al., in a prospective cohort study, reported that at two years of corrected age, a positive effect of the early introduction of complementary foods on length and weight: very low birth weight infants were on average ~0.4 cm taller (95% CI −0.1 to −0.6) and 100 g heavier (95% CI −0.02 to −0.2) for each month of earlier introduction of complementary food [25]. A positive association between weaning before four months of corrected age and weight gain at 18 to 24 months of corrected age, in very preterm infants born with a gestational age <32 weeks, was reported by Rodriguez et al. [26]. Instead, Gupta et al. reported similar growth parameters at 12 months of corrected age in preterm infants (<34 weeks of gestation) randomized to start weaning either at 4 or 6 months of corrected age [27]. They have also investigated motor and mental development, lipid profile, insulin resistance, blood pressure and serum ferritin showing no differences among groups. However, it must be considered the work of Gupta et al. was conducted in a low-income country and, as a result, their findings may not be applicable to different situations [27]. Morgan et al. evaluated two trials of preterm infants (<37 weeks of gestational age), weaned either early (≤12 weeks) or late (>12 weeks), reporting comparable growth rates between 3 and 9 months post-term; moreover, the attained weight and length at 18 months post-term did not differ between both groups [28]. A systematic review of five studies was not able to perform a meta-analysis, because the outcomes evaluated were not comparable [14]. Two studies evaluated body mass index (BMI): one did not report any significant difference of BMI index *Z*-score at 1 year between early- and late-weaned infants [27], whereas the other study concluded that, at 1 year of age, the risk of higher BMI was lower when weaning started ≤4 months of age [29]. Morgan et al. found a greater gain in the subscapular skinfold thickness between 3 and 9 months post-term in the early weaned group (before 12 weeks post term) [28]. At the moment, no clear conclusion can be drawn on the relationship between weaning and infants’ later risk of overweight and obesity.

## Conclusions

The increased nutritional needs of preterm infants should be adequately met for storage and to maintain and support catch-up growth during the first year of life [11]. The lack of scientific evidence regarding the optimal age and content of weaning result in different, sometime conflicting, indications by caregivers, often leaving parents and caregivers alone and confused. According to the available data, there is no definite positive effect of early weaning on infant growth and nutritional outcomes, but, at the same time, early weaning seems to not be associated with an increased risk of overweight/obesity later in life.
